# Burden of Illness in UK Subjects with Reported Respiratory Infections Vaccinated or Unvaccinated against Influenza: A Retrospective Observational Study

**DOI:** 10.1371/journal.pone.0134928

**Published:** 2015-08-19

**Authors:** Rhys D. Pockett, John Watkins, Phil McEwan, Genevieve Meier

**Affiliations:** 1 Swansea Center for Health Economics, Swansea University, SA2 8PP, Wales, United Kingdom; 2 College of Biomedical and Life Sciences, Cardiff University, Cardiff, CF14 4YS, Wales, United Kingdom; 3 Health Economics, GSK Vaccines, 1300, Wavre, Belgium; National Center for Immunization and Respiratory Diseases, UNITED STATES

## Abstract

**Objective:**

Detailed data are lacking on influenza burden in the United Kingdom (UK). The objective of this study was to estimate the disease burden associated with influenza-like illness (ILI) in the United Kingdom stratified by age, risk and influenza vaccination status.

**Methods:**

This retrospective, cross-sectional, exploratory, observational study used linked data from the General Practice Research Database and the Hospital Episode Statistics databases to estimate resource use and cost associated with ILI in the UK.

**Results:**

Data were included from 156,193 patients with ≥1 general practitioner visit with ILI. There were 21,518 high-risk patients, of whom 12,514 (58.2%) were vaccinated and 9,004 (41.8%) were not vaccinated, and 134,675 low-risk patients, of whom 17,482 (13.0%) were vaccinated and 117,193 (87.0%) were not vaccinated. High-risk vaccinated patients were older (p<0.001) and had more risk conditions (p<0.001). High-risk (odds ratio [OR] 2.16) or vaccinated (OR 1.19) patients had a higher probability of >1 general practitioner visit compared with low-risk and unvaccinated patients. Patients who were high-risk and vaccinated had a reduced risk of >1 general practitioner visit (OR 0.82; p<0.001). High-risk individuals who were also vaccinated had a lower probability of ILI-related hospitalisation than individuals who were high-risk or vaccinated alone (OR 0.59). In people aged ≥65 years, the mortality rate was lower in vaccinated than unvaccinated individuals (OR 0.75). The cost of ILI-related GP visits and hospital admissions in the UK over the study period in low-risk vaccinated patients was £27,391,142 and £141,932,471, respectively. In low-risk unvaccinated patients the corresponding values were £168,318,709 and £112,534,130, respectively.

**Conclusions:**

Although vaccination rates in target groups have increased, many people are still not receiving influenza vaccination, and the burden of ILI in the United Kingdom remains substantial. Improving influenza vaccination uptake may have the potential to reduce this burden.

## Introduction

Influenza is an acute self-limiting viral disease which, in the majority of cases, is inconvenient but generally not serious. However, seasonal influenza can cause severe illness, hospitalisations and deaths, especially in those who are at ‘high risk’ due to age or underlying chronic health problems.

There are three types of influenza virus, Type A, Type B and Type C [1]; only A and B cause significant disease in humans [2]. Influenza A, of which there are currently two subtypes circulating, H1N1 and H3N2 [3], is responsible for seasonal epidemic and occasional pandemic activity. Influenza B, which currently has two antigenically distinct lineages, Yamagata and Victoria, is responsible for outbreaks particularly in institutional settings.

Influenza can be prevented by vaccination. Annual vaccination against seasonal influenza is recommended for high-risk groups, including people aged 65 years or more and people with chronic health conditions [4], and the United Kingdom (UK) recently extended vaccination recommendations to include children [5,6].

Although several studies have previously estimated the burden of illness attributable to influenza in the UK [7–9], they have some limitations. For example, one did not include hospital data and thus is expected to have underestimated the burden of influenza [7], and others did not link hospital and general practice databases [8,9] or collect vaccination and risk status [8]. There is considerable disparity in estimates of influenza mortality derived from previous studies [10]. Furthermore, data are lacking on the burden of illness stratified by risk status, complication status (with or without complications of influenza recorded) or vaccination status. A recent review of cost-of-illness studies in influenza worldwide identified no UK studies reporting national cost-of-illness estimates, and only a few studies on the cost of influenza in specific population groups (children) [11]. Thus, although the burden of influenza in England has been shown to be substantial, accounting for 11,199 hospital admissions and 400 deaths annually in patients not in a clinical risk group, and 6,054 hospital admissions and 1,406 deaths annually in patients in a clinical risk group [9], it has not been described in sufficient detail.

To provide a more detailed understanding of the burden of influenza-like illness (ILI) to healthcare resources in the UK, we have conducted a large database analysis using linked hospital and general practice databases to quantify primary and secondary resource use and costs associated with ILI. To our knowledge, this is the first study to use linked hospital and general practice databases to assess the burden of illness in both primary and secondary care in ILI in the UK. The primary results of the study have been published elsewhere (Watkins et al., in preparation). The objective of the present analysis was to examine resource use and costs by vaccination and risk status.

## Materials and Methods

This was a retrospective, cross-sectional, observational study using data extracted from UK databases for the period 21 January 2001 to 31 March 2009 inclusive. The study period ended immediately before the 2009 H1N1 influenza pandemic. The data were divided into influenza seasons running from 1 April to 31 March inclusive, e.g. 1 April 2001 to 31 March 2002, etc. The study thus covered nine influenza seasons. The first season (2000–2001) ran from the study start date on 1 January 2001 to 31 March 2001. Influenza circulated from week 4 in 2001 according to surveillance data from the Health Protection Agency (HPA), so the study would have captured the majority of the influenza cases in the 2000–2001 season, although it is possible that there were some cases in the last weeks of 2000 for which data were not collected.

### Data collection

Influenza cases are generally not virologically confirmed in routine UK clinical practice [8], and influenza or ILI is rarely recorded specifically in medical notes [12]. Thus, neither a specific diagnosis of influenza/ILI nor virologically confirmed influenza could be used as the selection method. The General Practice Research Database (GPRD) codes general practitioner (GP) consultations using clinical READ codes. The proportion of patients in the GPRD with linked data in the Hospital Episode Statistics (HES) dataset is 58.8%, and the linked patients are representative of the whole GPRD population [13]. Since this study was initiated, the GPRD has changed its name and is now known as The Clinical Practice Research Datalink (CPRD).

Data on patients who had at least one GP consultation with a READ code describing influenza, acute upper or lower respiratory tract infection, or ILI were extracted from the linked GPRD-HES dataset for the period between 21^st^ January 2001 and 31^st^ March 2009 inclusive. Consultations coded with any of these READ codes that occurred during periods of peak influenza circulation were attributed to ILI (S1 File). Consultations coded with any of these specific READ codes that occurred during periods of peak influenza circulation were attributed to ILI. Peak influenza periods were those weeks when influenza A and influenza B were circulating, identified by regression analysis of surveillance data on circulating influenza and other respiratory pathogens obtained from the UK Health Protection Agency (HPA) for the period November 2002 to December 2008 [14], using the same technique as Pitman et al. [8] (described further below). Patients with GP consultations coded with a relevant READ code during these influenza periods were included in the study if they also had a minimum of 14 days observation before and after the index event; and at least 1 year observation before diagnosis.

Patients were stratified by age group (<5 years; 5–18 years, 19–49 years, 50–64 years, 65+ years), by risk status (high-risk as defined by National Institute for Health and Care Excellence [15] or Department of Health [16] criteria, or low-risk) and by influenza vaccination status. Patients were categorised as ‘vaccinated’ if they had a record of an influenza vaccination in the same winter flu season as the index episode.

Data on GP consultations and hospital admissions were extracted from the linked GPRD and HES dataset (S1 File). If there were two GP consultations recorded for a patient on the same day, only one was counted. Data were observed for 14 days after the GP consultation to identify any hospital admission for influenza or complications following influenza. Only the first episode of each hospitalisation was retained in the dataset, as this was more likely to be related to the cause of the admission rather than to subsequent complications.

Mortality data were identified from the linked GPRD-HES dataset, where mortality was recorded by the GP or at hospital discharge. Population estimates were obtained from the Office for National Statistics (ONS), using mid-year estimates for the resident population for 2001–2009 [17].

Surveillance data on circulating influenza and other respiratory infections were obtained from the UK HPA for the period November 2002 to December 2008, which were grouped into flu weeks (periods of approximately 4–5 weeks). Data on the mismatch between the influenza vaccine and circulating influenza virus strains/lineages were obtained from the HPA [14].

### Outcomes

The primary outcome measures were mortality, resource use (only those GP consultations and hospital admissions with READ codes related to the episode were included) and cost associated with influenza in primary and secondary care, stratified by age group, complication status, vaccination status and risk status. This paper presents data on vaccination uptake, and resource use, cost and mortality by vaccination status and degree of mismatch. Other results of the study have been published elsewhere (Watkins et al., in preparation). Medications prescribed were limited to include items such as antipyretics, analgesics, antivirals, nasal decongestants, antihistamines and antibiotics. Furthermore, only those encounters related with an episode were included in the analysis any routine visits or unrelated encounters were excluded.

### Cost data

Unit costs for hospitalisations, GP consultations and outpatient visits were obtained from the Personal Social Services Research Unit database for 2011 [18]. Resource use data were combined with unit cost data to estimate total costs, from the perspective of the UK National Health Service. All costs were expressed in £ (pounds sterling) using 2011 costs.

### Data analysis

Regression analysis was used to estimate the proportion of GP consultations, hospitalisations and deaths potentially attributable to each pathogen, using the same technique as Pitman et al. [8], previously described by Ryan et al [19].

The observed temporal trends in laboratory confirmed reports of agents that could potentially cause influenza/acute respiratory infection/ILI was compared with the number of events under consideration (GP consultations, hospital episodes, or mortality) over the same time period. The latter was used as the dependent variable of a multiple regression. The independent variables were the weekly number of national laboratory reports (HPA data, 2001–2009) for adenovirus, coronavirus, influenza A, influenza B, *Mycoplasma pneumoniae*, parainfluenza, respiratory syncytial virus, and rhinovirus. Laboratory reports were not age stratified as this would have resulted in too few individuals in certain classes.

This method of linear regression uses the number of laboratory reports for pathogen *i* in week *j*, *L*_*ij*_ and a background number of national laboratory reports to estimate the regression coefficients *α*_*j*_ that fit into the equation: *Y*_*j*_ = *c + Σ*_*i*_ *α*_*i*_ * L_*ij*_, *Y*_*j*_ indicating the independent variable (weekly number of national laboratory reports aggregated over all age groups). A multivariable regression, assuming normality of the error terms, was performed. The initial model included all the organisms as explanatory variables. The significance of each organism was assessed, and if the *p* value was >0.1 or the variable was significant but the resulting coefficient negative (which is biologically implausible) the variable was discarded from the model. The validity of the final model was assessed in terms of the proportion of the variation it explained (R^2^), the significance of the joint relationship between the observed and independent variables in each model (i.e. the probability that R differed significantly from zero), and the impact of changes in the model specification.

Regression analysis was used to assess the relationship between burden and the degree of mismatch between the influenza vaccine and the circulating influenza virus strains in each year. Multivariate analysis was used to uncover structure within the data and those factors contributing to excess burden. This was based on knowledge of the source data once it has been obtained and processed. Generalised linear models were fitted to characterise any association between baseline characteristics and burden

Vaccinated and unvaccinated patients categorised as high-risk were compared using an independent samples t-test with age and number of risk conditions as the test variables. A similar analysis was undertaken in patients categorised as not high-risk, using age as the test variable.

To extrapolate from the database sample to the total UK population, a multiplying factor was calculated. The annual average total UK population during the study period was 60,313,633, using ONS mid-year estimates for the resident population for 2001–2009 [17]. The annual average number of patients in the linked GPRD-HES database population was 1,662,953 between 2000 and 2009, calculated from the GPRD-HES data extract. Dividing the database population by the total UK population gives a multiplying factor of 36.3. Results from the database were then multiplied by this factor to estimate the total burden in the whole of the UK. The same factor was used for all patient groups, as age-specific, complication-specific and risk-specific populations were not available. No adjustments for demographics were made.

## Results

### Study population

The linked dataset included 156,193 patients with at least one GP consultation for ILI (defined as described in the Methods). The mean age was 42.7 years and 55% were women. The demographic characteristics of the study population have been published elsewhere (Watkins et al., in preparation). Table 1 shows the breakdown of general physician visit by READ code.

**Table 1:** General Practitioner Visit Break down by READ Code.

READ Code	Count of patient visits	Description
H27z.11	125769	Flu like illness
H27..00	14359	Influenza
H2z..00	5789	Pneumonia or influenza NOS
16L..00	3809	Influenza-like symptoms
H27z.12	2050	Influenza like illness
H27z.00	1937	Influenza NOS
H2…00	1805	Pneumonia and influenza
H2y..00	203	Other specified pneumonia or influenza
H27y100	151	Influenza with gastrointestinal tract involvement
H271z00	101	Influenza with respiratory manifestations NOS
H270000	68	Influenza with bronchopneumonia
H271000	66	Influenza with laryngitis
H271100	31	Influenza with pharyngitis
H270.00	21	Influenza with pneumonia
H270.11	15	Chest infection—influenza with pneumonia
H270z00	11	Influenza with pneumonia NOS
H271.00	6	Influenza with other respiratory manifestation
F030800	1	Encephalitis due to influenza-specific virus not identified
H27y000	1	Influenza with encephalopathy

### Vaccination uptake

Table 2 shows the percentage of patients in each risk group vaccinated by year. Elderly patients (age 65+ years) had the highest uptake rates, and uptake rates generally increased between the beginning and end of the study period in each risk group.

**Table 2:** Percentage of patients with a vaccination record each year, by risk group.

Year	All High Risk	Respiratory	Central Nervous System	Diabetes	Chronic Heart Disease	Liver Disease	Renal Disease	Compromised Immune System	Age 65+ years	Not high risk and aged <65 years
2001	43.6%	40.5%	40.7%	55.7%	49.2%	32.8%	54.8%	52.6%	65.4%	14.3%
2002	48.2%	43.1%	44.8%	60.5%	52.2%	35.3%	58.9%	55.8%	70.9%	15.0%
2003	51.5%	47.6%	48.4%	67.3%	57.7%	35.3%	65.3%	59.9%	76.0%	15.3%
2004	55.9%	51.1%	52.4%	74.3%	61.1%	40.0%	68.4%	67.1%	79.4%	15.3%
2005	61.4%	53.2%	54.5%	80.5%	67.5%	47.7%	72.6%	73.3%	84.5%	17.4%
2006	58.1%	64.7%	50.5%	77.8%	64.7%	50.0%	70.4%	66.4%	81.3%	15.0%
2007	59.1%	51.3%	54.2%	80.2%	65.6%	52.2%	72.3%	69.9%	80.3%	14.8%
2008	59.4%	51.6%	55.8%	79.2%	65.7%	53.7%	74.8%	71.3%	78.4%	15.0%
2009	61.2%	54.0%	56.0%	80.2%	67.6%	62.5%	77.6%	68.8%	75.9%	18.3%

Table 3 shows the percentage of patients vaccinated each year by age and risk status. Overall, there were 21,518 high-risk patients, of whom 12,514 (58.2%) were vaccinated and 9,004 (41.8%) were not vaccinated. There were 134,675 low-risk patients of whom 17,482 (13.0%) were vaccinated and 117,193 (87.0%) were not vaccinated.

**Table 3:** Vaccination status by age, risk group and year.

Influenza Year (April—March)				Non-vaccinated		Vaccinated	
	Age group	Risk status	Total N	N	%	N	%
2001	Overall		18,591	14,903	80.2%	3,688	19.8%
	<5 years	Not At Risk	373	368	98.7%	5	1.3%
		At Risk	30	29	96.7%	1	3.3%
	5–18 years	Not At Risk	2,095	2,065	98.6%	30	1.4%
		At Risk	145	122	84.1%	23	15.9%
	19–49 years	Not At Risk	8,821	8,474	96.1%	347	3.9%
		At Risk	651	481	73.9%	170	26.1%
	50–64 years	Not At Risk	2,887	2,352	81.5%	535	18.5%
		At Risk	570	291	51.1%	279	48.9%
	>64 years	Not At Risk	2,104	515	24.5%	1,589	75.5%
		At Risk	915	206	22.5%	709	77.5%
2002	Overall		16,110	13,384	83.1%	2,726	16.9%
	<5 years	Not At Risk	400	399	99.8%	1	0.3%
		At Risk	25	25	100.0%	0	0.0%
	5–18 years	Not At Risk	1,973	1,952	98.9%	21	1.1%
		At Risk	111	91	82.0%	20	18.0%
	19–49 years	Not At Risk	7,485	7,213	96.4%	272	3.6%
		At Risk	578	423	73.2%	155	26.8%
	50–64 years	Not At Risk	2,658	2,291	86.2%	367	13.8%
		At Risk	557	269	48.3%	288	51.7%
	<64 years	Not At Risk	1,529	519	33.9%	1,010	66.1%
		At Risk	794	202	25.4%	592	74.6%
2003	Overall		23,734	19,930	84.0%	3,804	16.0%
	<5 years	Not At Risk	675	674	99.9%	1	0.1%
		At Risk	28	27	96.4%	1	3.6%
	5–18 years	Not At Risk	2,979	2,932	98.4%	47	1.6%
		At Risk	157	128	81.5%	29	18.5%
	19–49 years	Not At Risk	10,909	10,568	96.9%	341	3.1%
		At Risk	816	607	74.4%	209	25.6%
	50–64 years	Not At Risk	4,194	3,666	87.4%	528	12.6%
		At Risk	779	404	51.9%	375	48.1%
	<64 years	Not At Risk	2,029	672	33.1%	1,357	66.9%
		At Risk	1,168	252	21.6%	916	78.4%
2004	Overall		13,159	10,256	77.9%	2,903	22.1%
	<5 years	Not At Risk	193	192	99.5%	1	0.5%
		At Risk	13	13	100.0%	0	0.0%
	5–18 years	Not At Risk	1,055	1,032	97.8%	23	2.2%
		At Risk	51	36	70.6%	15	29.4%
	19–49 years	Not At Risk	6,070	5,817	95.8%	253	4.2%
		At Risk	534	339	63.5%	195	36.5%
	50–64 years	Not At Risk	2,396	2,029	84.7%	367	15.3%
		At Risk	529	210	39.7%	319	60.3%
	<64 years	Not At Risk	1,414	391	27.7%	1,023	72.3%
		At Risk	904	197	21.8%	707	78.2%
2005	Overall		20,905	16,678	79.8%	4,227	20.2%
	<5 years	Not At Risk	271	265	97.8%	6	2.2%
		At Risk	8	8	100.0%	0	0.0%
	5–18 years	Not At Risk	1,948	1,910	98.0%	38	2.0%
		At Risk	122	87	71.3%	35	28.7%
	19–49 years	Not At Risk	9,877	9,491	96.1%	386	3.9%
		At Risk	818	530	64.8%	288	35.2%
	50–64 years	Not At Risk	3,843	3,289	85.6%	554	14.4%
		At Risk	806	351	43.5%	455	56.5%
	<64 years	Not At Risk	1,930	484	25.1%	1,446	74.9%
		At Risk	1,282	263	20.5%	1,019	79.5%
2006	Overall		18,015	14,299	79.4%	3,716	20.6%
	<5 years	Not At Risk	266	264	99.2%	2	0.8%
		At Risk	5	5	100.0%	0	0.0%
	5–18 years	Not At Risk	2,520	2,453	97.3%	67	2.7%
		At Risk	133	94	70.7%	39	29.3%
	19–49 years	Not At Risk	8,108	7,707	95.1%	401	4.9%
		At Risk	634	397	62.6%	237	37.4%
	50–64 years	Not At Risk	3,085	2,587	83.9%	498	16.1%
		At Risk	659	257	39.0%	402	61.0%
	<64 years	Not At Risk	1,510	344	22.8%	1,166	77.2%
		At Risk	1,095	191	17.4%	904	82.6%
2007	Overall		18,960	15,214	80.2%	3,746	19.8%
	<5 years	Not At Risk	224	222	99.1%	2	0.9%
		At Risk	8	6	75.0%	2	25.0%
	5–18 years	Not At Risk	1,755	1,710	97.4%	45	2.6%
		At Risk	89	60	67.4%	29	32.6%
	19–49 years	Not At Risk	9,257	8,912	96.3%	345	3.7%
		At Risk	664	435	65.5%	229	34.5%
	50–64 years	Not At Risk	3,407	2,951	86.6%	456	13.4%
		At Risk	762	315	41.3%	447	58.7%
	<64 years	Not At Risk	1,454	354	24.3%	1,100	75.7%
		At Risk	1,340	249	18.6%	1,091	81.4%
2008	Overall		20,743	16,863	81.3%	3,880	18.7%
	<5 years	Not At Risk	244	243	99.6%	1	0.4%
		At Risk	8	8	100.0%	0	0.0%
	5–18 years	Not At Risk	1,523	1,488	97.7%	35	2.3%
		At Risk	66	43	65.2%	23	34.8%
	19–49 years	Not At Risk	10,223	9,870	96.5%	353	3.5%
		At Risk	670	441	65.8%	229	34.2%
	50–64 years	Not At Risk	4,145	3,679	88.8%	466	11.2%
		At Risk	783	342	43.7%	441	56.3%
	<64 years	Not At Risk	1,752	478	27.3%	1,274	72.7%
		At Risk	1,329	271	20.4%	1,058	79.6%
2009	Overall		5,976	4,670	78.1%	1,306	21.9%
	<5 years	Not At Risk	71	71	100.0%	0	0.0%
		At Risk	3	3	100.0%	0	0.0%
	5–18 years	Not At Risk	497	486	97.8%	11	2.2%
		At Risk	22	15	68.2%	7	31.8%
	19–49 years	Not At Risk	2,626	2,540	96.7%	86	3.3%
		At Risk	165	90	54.5%	75	45.5%
	50–64 years	Not At Risk	1,232	1,080	87.7%	152	12.3%
		At Risk	255	98	38.4%	157	61.6%
	<64 years	Not At Risk	668	194	29.0%	474	71.0%
		At Risk	437	93	21.3%	344	78.7%

In the high-risk group, the mean age of the vaccinated group was 65.1 years (standard deviation [SD] 16.5) compared with 48.2 years (SD 20.4) in the unvaccinated group (p<0.001). The mean number of risk conditions in the vaccinated group was 1.29 (SD 0.60), compared with 1.15 (SD 0.45) in the unvaccinated group (p<0.001). In the patients without risk conditions, vaccinated patients were also older than unvaccinated patients (mean 64.6 years [SD 17.3] compared with mean 36.6 years [SD 16.8], p = 0.003). Vaccinated patients were therefore statistically significantly different from unvaccinated patients, as they were older and had more risk conditions. This should be taken into account when considering outcomes in the different groups.

### Resource use in low-risk patients

Table 4 shows the number and cost of ILI-related hospital admissions, GP consultations and outpatient visits in low-risk patients by vaccination and complication status. Data on the absolute numbers of ILI-related admissions, GP consultations and outpatient visits can be found in S1 Table, and data on hospital admissions stratified by the route of admission (via accident and emergency, GP referral or other routes) can be found in S2 Table.

**Table 4:** Resource use in low-risk patients.

	Vaccinated						Non-Vaccinated					
	Overall Influenza		Influenza with complications recorded		Influenza without complications recorded		Overall Influenza		Influenza with complications recorded		Influenza without complications recorded	
	N	%	N	%	N	%	N	%	N	%	N	%
**Inpatient Admissions (All)**												
Had ≥ 1 hospital admission	342	2.0%	342	2.0%	0	0.0%	534	0.5%	525	0.4%	9 *	0.0%
Had ≥ 1 hospital admission (UK)	12,404		12,404		0		19,368		19,041		326	
Mean number of unique admissions (SD)	1.2 (0.50)		1.2 (0.50)		-	-	1.1 (0.28)		1.1 (0.28)		1 (0.0)	
Mean length of stay (SD)	13.9 (16.7)		13.9 (16.7)		-	-	7.7 (10.6)		7.8 (10.6)		3.1 (3.2)	
Mean total cost (SD)	£3,913,328 (£1,959,010)		£3,913,328 (£1,959,010)		-	-	£3,102,764 (£1,087,250)		£3,090,087 (£1,068,925)		£19,139 (£0)	
Mean total cost (SD) (UK)	£141,932,471 (£71, 051,323)		£141,932,471 (£71, 051,323)		-	-	£112,534,130 (39,433,464)		£112,074,348 (££38,768,835)		££694,152 (£0)	
**GP Surgery Visits**												
Had ≥ 1 GP surgery visit*	17,482	100%	1,334	7.6%	16,148	92.4%	117,193	100%	6,032	5.1%	111,161	94.9%
Had ≥ 1 GP surgery visit (UK)	634,055		48,383		585,672		4,250,472		218,775		4,031,698	
Mean number of GP Surgery visits (SD)	1.2 (0.46)		1.9 (0.84)		1.1 (0.36)		1.1 (0.38)		1.9 (0.74)		1.1 (0.29)	
Mean (SD) total costs	£755,222 (£289,502)		£91,246 (£40,340)		£639,461 (£209,278)		£4,640,843 (£1,603,200)		£412,589 (£160,692)		£4,401,976 (£1,160,521)	
Mean (SD) total costs (UK)	£27,391,142 (£10,499,946)		£3,309,401 (£1,463,091)		£23,192,607 (£7,590,303)		£168,318,709 (£58,146,452)		£14,964,188 (£5,828,137)		£159,655,243 (£42,090,930)	
**GP Prescriptions**												
Had ≥ 1 prescription	12,761	73.0%	1,103	6.3%	11,658	66.7%	52,591	44.9%	5,133	4.4%	47,458	40.5%
Had ≥ 1 prescription (UK)	462,829		40,005		422,824		1,907,423		186,169		1,721,254	
Mean number of prescriptions obtained (SD)	3.2 (2.9)		3.7 (3.3)		3.2 (2.8)		1.6 (1.4)		1.8 (1.4)		1.6 (1.4)	
Average cost per script	£1.01 (£3.96)		£1.17 (£4.05)		£0.98 (£3.95)		£0.61 (£2.26)		£0.63 (£2.01)		£0.60 (£2.29)	
**Out Patient Clinic Care **												
Had ≥ 1 OP Clinic visit	91	0.5%	31	0.2%	60	0.3%	303	0.3%	90	0.1%	213	0.2%
Had ≥ 1 OP Clinic visit (UK)	3,300		1,124		2,176		10,990		3,264		7,725	
Mean number of OP clinic visit(SD)	1.0 (0.21)		1.1 (0.25)		1.0 (0.28)		1.1 (0.27)		1.1 (0.25)		1.1 (0.27)	
Mean (SD) total costs	£13,377 (£2,809)		£5,013 (£1,139)		£8,820 (£2,470)		£48,995 (£12,026)		£14,553 (£3,308)		£34,442 (£8,454)	
Mean (SD) total costs (UK)	£485,170 (££101,880)		£181,816 (£41,310)		£319,893 (£89,584)		£1,776,999 (£436,171)		£527,823 (£119,978)		£1,249,177 (£306,618)	

GP, general practitioner; OP, outpatient; SD, standard deviation; UK, extrapolated to UK population

*Review of the records indicated that these patients had been mis-coded and had complications

In low-risk patients, unvaccinated patients used more GP and outpatient resources than vaccinated patients, reflecting the large number of unvaccinated low-risk cases in the database. Secondary care resource use was more evenly split, with slightly higher total resource use and cost in the group of vaccinated patients than in the group of unvaccinated patients. This is consistent with the older age of vaccinated patients compared with the unvaccinated group. Extrapolated to the UK, the cost of ILI-related GP visits and hospital admissions over the study period in low-risk vaccinated patients was £27,391,142 and £141,932,471, respectively. In low-risk unvaccinated patients the corresponding values were £168,318,709 and £112,534,130, respectively.

Secondary care resource use was concentrated in cases of influenza with complications recorded, regardless of vaccination status.

### Resource use in high-risk patients

Table 5 shows the number and cost of ILI-related hospital admissions, GP consultations and outpatient visits in high-risk patients by vaccination and complication status. Data on the absolute numbers of ILI-related admissions, GP consultations and outpatient visits can be found in S1 Table, and data on hospital admissions stratified by the route of admission (via accident and emergency, GP referral or other routes) can be found in S3 Table.

**Table 5:** Resource use in high-risk patients.

	Vaccinated						Non-Vaccinated					
	Overall Influenza		Influenza with complications recorded		Influenza without complications recorded		Overall Influenza		Influenza with complications recorded		Influenza without complications recorded	
	N	%	N	%	N	%	N	%	N	%	N	%
**Inpatient Admissions (All)**												
Had ≥ 1 hospital admission	503	4.0%	503	4.0%	0	0.0%	222	2.5%	219	2.4%	3 *	0.0%
Had ≥ 1 hospital admission (UK)	18,243		18,243		0		8,052		7,943		109	
Mean number of unique admissions (SD)	1.2 (0.53)		1.2 (0.53)		-	-	1.2 (0.51)		1.2 (0.51)		1 (0.0)	
Mean length of stay (SD)	12.9 (5.9)		12.9 (5.9)		-	-	12.4 (19.5)		12.6 (19.6)		4 (3.6)	
Mean total cost (SD)	£5,341,498 (£1,078,996)		£5,341,498 (£1,078,996)		-	-	£2,266,105 (£1,514,544)		£2,271,538 (£1,501,739)		£8,232 (£0)	
Mean total cost (SD) (UK)	£193,730,761 (£39,134,100)		£193,730,761 (£39,134,100)		-	-	£82,189,349 (£54,930,988)		£82,386,399 (£54,466,563)		£298,566 (£0)	
**GP Surgery Visits**												
Had ≥ 1 GP surgery visit	12,514	100%	1,807	14.4%	10,707	85.6%	9,004	100%	1,225	13.6%	7,779	86.4%
Had ≥ 1 GP surgery visit (UK)	453,870		65,538		388,332		326,566		44,430		282,137	
Mean number of GP Surgery visits (SD)	1.3 (0.65)		1.9 (1.0)		1.2 (0.49)		1.3 (0.62)		1.8 (0.98)		1.2 (0.49)	
Mean (SD) total costs	£585,655 (£292,828)		£123,599 (£65,052)		£462,542 (£188,871)		£421,387 (£200,969)		£79,380 (£43,218)		£336,053 (£137,222)	
Mean (SD) total costs (UK)	£21,241,118 (£10,620,577)		£4,482,811 (£2,359,371)		£16,775,933 (£6,850,161)		£15,283,283 (£7,288,944)		£2,879,033 (£1,567,473)		£12,188,304 (£4,976,904)	
**GP Prescriptions**												
Had ≥ 1 prescription	10,454	83.5%	1,454	11.6%	9,000	71.9%	6,398	71.1%	985	10.9%	5,413	60.1%
Had ≥ 1 prescription (UK)	379,156		52,735		326,421		232,049		35,725		196,324	
Mean number of prescriptions obtained (SD)	5.6 (5.1)		6.1 (6.1)		5.5 (4.9)		3.6 (3.9)		3.6 (4.1)		3.6 (3.9)	
Average cost per script	£1.28 (£4.50)		£1.95 (£5.64)		£1.13 (£4.18)		£1.19 (£4.33)		£1.47 (£4.53)		£1.12 (£4.27)	
**Out Patient Clinic Care **												
Had ≥ 1 OP Clinic visit	106	0.8%	42	0.3%	64	0.5%	74	0.8%	29	0.3%	45	0.5%
Had ≥ 1 OP Clinic visit (UK)	3,845		1,523		2,321		2,684		1,052		1,632	
Mean number of OP clinic visit(SD)	1.1 (0.31)		1.1 (0.33)		1.1 (0.29)		1.1 (0.7)		1.0 (0.0)		1.2 (0.91)	
Mean (SD) total costs	£17,140 (£4,830)		£6,791 (£2,037)		£10,349 (£2,728)		£11,966 (£7,615)		£4,263 (£0)		£7,938 (£6,020)	
Mean (SD) total costs (UK)	£621,651 (£175,179)		£246,303 (£73,880)		£375,348 (£98,942)		£433,995 (£276,188)		£154,615 (£0)		£287,903 (£218,339)	

GP, general practitioner; OP, outpatient; SD, standard deviation; UK, extrapolated to UK population

*Review of the records indicated that these patients had been mis-coded and had complications

In high-risk patients, the group of vaccinated patients used more resources in primary and secondary care, compared with the group of unvaccinated patients. This is consistent with the older age and higher number of risk conditions in vaccinated than unvaccinated patients. As was the case in low-risk patients, secondary care resource use was concentrated in cases of influenza with complications recorded, regardless of vaccination status. Extrapolated to the UK, the cost of ILI-related GP visits and hospital admissions over the study period in high-risk vaccinated patients was £21,241,118 and £193,730,761, respectively. In high-risk unvaccinated patients the corresponding values were £15,283,283 and £82,189,349, respectively.

### Mortality

Table 6 shows the number of ILI-related deaths by vaccination status. Overall, more deaths were observed in vaccinated than unvaccinated patients. This is consistent with the systematic difference between vaccinated and unvaccinated individuals, as vaccinated individuals were older and more fragile with more risk conditions than unvaccinated individuals. However, among the group aged 65 years or more, the mortality rate among vaccinated individuals was 25% (odds ratio [OR] 0.75; 95% confidence interval [CI] 0.65 to 0.88; p<0.001) lower than in unvaccinated individuals (3.3% of the age category and 4.3% of the age category, respectively) (Table 6).

**Table 6:** Mortality by vaccination status and age.

Parameter	All			Influenza without complications recorded			Influenza with complications recorded		
	N	Deaths	Mortality %	N	Deaths	Mortality %	N	Deaths	Mortality %
**Vaccinated**									
All Patients	29,996	633	2.1%	26,855	434	1.6%	3,141	199	6.3%
All Patients (UK)	1,087,925	22,958		974,004	15,741		113,921	7,218	
Mean Age (SD)	64.8			64.5 (16.9)			67.1 (17.4)		
Age Categories									
<5	23	0	0.0%	20	0	0.0%	3	0	0.0%
5–18	537	1	0.2%	481	1	0.2%	56	0	0.0%
19–49	4,571	16	0.4%	4,153	16	0.4%	418	0	0.0%
50–64	7,086	32	0.5%	6,456	19	0.3%	630	13	2.1%
65+	17,779	584	3.3%	15,745	398	2.5%	2,034	186	9.1%
**Unvaccinated**									
All Patients	126,197	549	0.4%	118940	456	0.4%	7,257	93	1.3%
All Patients (UK)	4,577,038	19,912		4,313,834	16,539		263,204	3,373	
Mean Age (SD)	37.5			37.4 (17.2)			39.3 (19.3)		
Age Categories									
<5	2,822	9	0.3%	2,585	9	0.3%	237	0	0.0%
5–18	16,704	28	0.2%	15,827	28	0.2%	877	0	0.0%
19–49	74,335	184	0.2%	70,327	177	0.3%	4,008	7	0.2%
50–64	26,461	74	0.3%	24,934	62	0.2%	1,527	12	0.8%
65+	5,875	254	4.3%	5,267	180	3.4%	608	74	12.2%

SD, standard deviation; UK, extrapolated to UK population

### Mismatch analysis

Table 7 shows the circulating virus strains, influenza B vaccine lineages, and the degree of matching between the vaccine and the circulating B lineage in each year of the study, from HPA data. There was a mismatch between the vaccine and circulating influenza B virus lineage in five of the ten years, and the two influenza B lineages co-circulated in three of the ten years.

**Table 7:** Mismatch between vaccine lineages and circulating influenza B lineages (HPA data).

	Distribution A & B		Vaccine lineage	Distribution within B		Matching within B
Season	Influenza A	Influenza B	B-lineage	Victoria	Yamagata	
2000–2001	37%	63%	Yamagata	0%	100%	100%
2001–2002	97%	3%	Yamagata	0%	100%	100%
2002–2003	51%	49%	Victoria	100%	0%	100%
2003–2004	99.6%	0%	Victoria	0%	100%	0%
2004–2005	85%	15%	Yamagata	17%	83%	83%
2005–2006	30%	70%	Yamagata	99%	1%	1%
2006–2007	99%	1%	Victoria	33%	67%	33%
2007–2008	64%	36%	Victoria	0%	100%	0%
2008–2009	90%	10%	Yamagata	94%	6%	6%
2009–2010	99%	1%	Victoria	100%	0%	100%
Average	75%	25%		44%	56%	52%

The season 2005–2006 also had a very high circulation of influenza B, with influenza B accounting for 70% of the circulating influenza that season.

**Fig 1:**
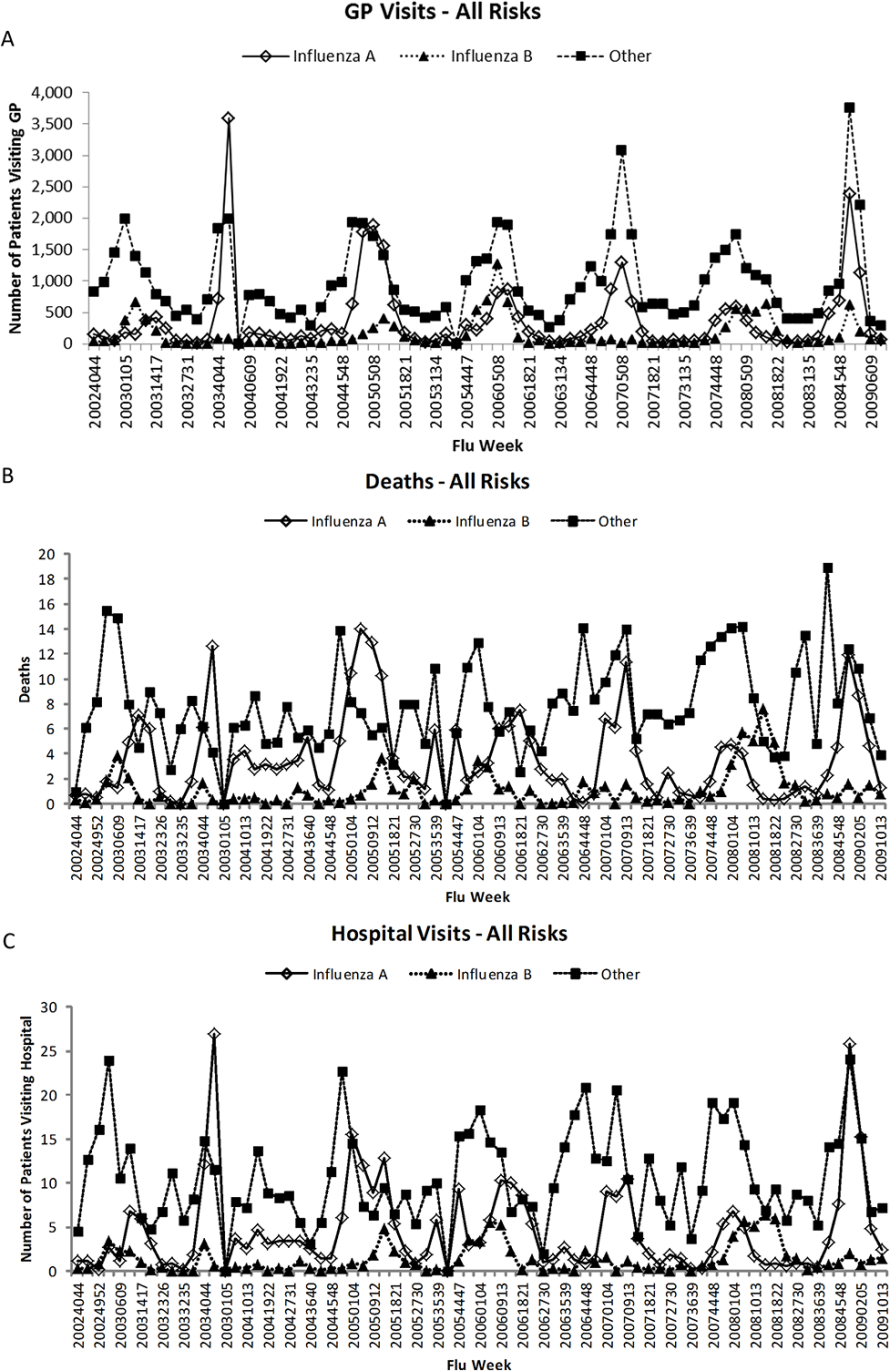
Number of (A) GP visits, (B) deaths and (C) hospitalisations by influenza week and pathogen. ‘Flu week’ indicates the year (first four digits) and range of weeks (last four digits), e.g. ‘20024044’ = 2002, weeks 40–44. GP, general practitioner.

There was a trend for influenza B mismatched seasons to be associated with peaks in ILI-related GP visits (Fig 1A). The season with highest mismatch and highest influenza B circulation, 2005–2006, was clearly associated with a peak in ILI-related GP visits (Fig 1A). The highest rate of ILI-related deaths occurred in 2007–2008 and the second highest in 2005–2006, both influenza B mismatched seasons (Fig 1B). ILI-related hospital admissions showed a similar pattern (Fig 1C).

Hospital admissions were highly correlated with GP visits (correlation coefficient 0.893 for influenza A, 0.789 for influenza B and 0.715 for others), and mortality was highly correlated with hospitalisation (correlation coefficient 0.884 for influenza B, 0.870 for influenza A, and 0.632 for others).

### Multivariate analysis

Table 8 shows the logistic regression coefficients for the likelihood of >1 GP visit, hospital admission and mortality. High-risk patients and patients who had been vaccinated had a higher probability of >1 ILI-related GP visit, compared with those who were not high-risk or had not been vaccinated. The OR for vaccinated patients was 1.19 (95% CI 1.11 to 1.28; p<0.001) and the OR for high-risk patients was 2.16 (95% CI 2.02 to 2.28; p<0.001). However, there was an interaction such that patients who were high-risk and vaccinated had a reduced risk of >1 GP visit, with an OR of 0.82 (95% CI 0.75 to 0.91; p<0.001). There was no apparent age interaction, so these effects were relevant for all age groups.

**Table 8:** Logistic regression coefficients for the risk of >1 general practitioner visit, hospitalisation and mortality.

All ages	Coefficient	SE	t value	Pr(*>|*t|)
**Likelihood of >1 GP visit in the 3 months after influenza diagnosis**				
(Intercept)	-2.280	0.078	-29.190	0.000
Age *≤* 1 year	-0.233	0.085	-2.730	0.006
Age 2–4 years	-0.315	0.078	-4.040	0.000
Age 5–18 years	-0.090	0.076	-1.180	0.238
Age 19–49 years	*Reference age group*			
Age 50–64 years	0.193	0.083	2.340	0.020
Age *≥* 65 years	0.063	0.083	0.750	0.451
Female	0.144	0.020	7.340	0.000
Manifested influenza	0.241	0.066	3.640	0.000
Hospital Event	1.262	0.063	20.010	0.000
High risk patient	0.764	0.031	24.570	0.000
Vaccinated	0.177	0.036	4.990	0.000
High risk & Vaccinated	-0.193	0.048	-4.030	0.000
%Adenovirus	0.386	0.057	6.740	0.000
%Parainfluenza	0.732	0.139	5.280	0.000
% influenza B	-0.302	0.082	-3.690	0.000
%Coronavirus	-2.808	1.001	-2.810	0.005
High risk & Vaccinated	-0.252	0.050	-5.030	0.000
**Likelihood of hospital admission during the study period**				
(Intercept)	-6.158	0.107	-57.680	0.000
Age *≤* 1 year	2.199	0.425	5.180	0.000
Age 2–4 years	1.799	0.201	8.930	0.000
Age 5–18 years	0.331	0.152	2.180	0.029
Age 19–49 years	*Reference age group*			
Age 50–64 years	0.699	0.102	6.850	0.000
Age *≥* 65 years	1.903	0.108	17.600	0.000
Female	-0.307	0.060	-5.140	0.000
Manifested influenza	1..2276	0.103	11.900	0.000
Vaccine B mismatch	0.186	0.058	3.200	0.001
High-risk	1.174	0.096	12.250	0.000
Vaccinated	0.613	0.100	6.110	0.000
High-risk & Vaccinated	-0.533	0.128	-4.160	0.000
% adenovirus	0.816	0.166	4.920	0.000
% parainfluenza	1.517	0.361	4.200	0.000
**Likelihood of death during the study period**				
(Intercept)	-5.094	0.392	-13.000	0.000
Age *≤* 1 year	-1.096	0.495	-2.210	0.027
Age 2–4 years	-0.497	0.392	-1.270	0.204
Age 5–18 years	-0.318	0.394	-0.810	0.419
Age 19–49 years	*Reference age group*			
Age 50–64 years	1.310	0.397	3.300	0.001
Age *≥* 65 years	2.822	0.389	7.250	0.000
Female	-0.200	0.070	-2.870	0.004
Manifested	1.234	0.107	11.540	0.000
High-risk	0.376	0.077	4.910	0.000
Vaccinated	-0.246	0.084	-2.940	0.003
Complications	0.970	0.083	11.690	0.000
% influenza A	-0.841	0.177	-4.750	0.000
% RSV	-1.051	0.214	-4.910	0.000

GP, general practitioner; RSV, respiratory syncytial virus, SE, standard error

High-risk individuals who were also vaccinated had a lower probability of ILI-related hospitalisation than individuals who were high-risk or vaccinated alone (OR = 0.59; 95% CI 0.46 to 0.75; p<0.001). In years with influenza B mismatch, there was a statistically significant increase in the odds of hospital admission (OR = 1.2; 95% CI 1.07 to 1.35; p<0.001). Patients who were high-risk had a higher probability of ILI-related death during the study period (OR = 1.46; 95% CI 1.25 to 1.69; p<0.001), and patients who were vaccinated had a lower probability of death during the study period (OR = 0.78; 95% CI 0.66 to 0.92; p<0.01).

## Discussion

The present study builds on previous investigations of the burden of ILI in the UK [7,8] in several ways. To our knowledge, this is the first time that linked primary care and hospital databases have been used to estimate the burden of ILI. Furthermore, this is the first study to report quantitative data for ILI burden by complication status (with complications recorded or without complications recorded), age, risk and vaccination status.

Influenza vaccination uptake increased over the study period, but vaccination still failed to reach 20–25% of people aged 65 years and older. In England, the target rate for influenza vaccination in 2011–2012 was 75% in people aged 65+ years and 60% in other at-risk groups, increasing to 75% in 2013/2014 [20]. Our study indicates that the vaccination uptake rates achieved in the elderly population were close to the target range. However, vaccination uptake rates in other at-risk groups were generally exceedingly low compared with the 2013/2014 target of 75%, except in a few conditions such as diabetes and renal disease. Improving vaccination uptake rates in younger age groups with risk conditions, and further improving vaccination uptake in the elderly, may have the potential to reduce the considerable burden of ILI in the UK. Our analysis demonstrates that vaccinated high-risk individuals have better outcomes than high-risk unvaccinated individuals.

Most ILI cases occurred in individuals who were not categorised as high-risk, and who were not vaccinated. Over 117,000 such patients visited a GP and 534 were admitted to hospital in the study population, at an estimated cost of over £4 million and over £3 million, respectively. Scaled up to the whole UK population, this would be equivalent to over £168 million for GP visits and over £112 million for hospital admissions. This raises the possibility that mass annual vaccination could potentially have medical and economic benefits, by reducing the burden of influenza cases in individuals who are not in groups currently targeted for vaccination. Universal influenza vaccination was introduced in Ontario, Canada, in 2000 and has been associated with reductions in influenza-related mortality and healthcare use, compared with targeted vaccination programmes in other provinces [21]. It has been suggested that mass vaccination could be cost-saving in the USA [22].

Vaccinated patients differed significantly from unvaccinated patients, being older and with more risk conditions. This pattern is well known [23], and is to be expected from current government policy, which targets elderly and/or high-risk groups for influenza vaccination. It is consistent with the observed pattern of higher resource use in vaccinated patients in the high-risk group in the present study; since vaccinated individuals were older and had more risk conditions than unvaccinated individuals, they are likely to have more fragile health and thus to need more healthcare. As vaccinated and unvaccinated groups are significantly different, outcomes cannot be directly compared and the data should be interpreted with caution. The only population in which vaccinated and unvaccinated groups could be compared is the sub-group aged 65 years and older, as according to current policy all individuals in this age group should receive seasonal influenza vaccination. Among this group, the ILI-related mortality rate among vaccinated individuals was 25% lower than the mortality rate among unvaccinated individuals. This is consistent with a protective effect of influenza vaccination. The effect size was similar to that reported by previous studies in this population group, which have reported reductions of 33–62% in hospitalisations, 27–39% in hospitalisations for acute and chronic respiratory illness and 39–54 in all-cause mortality [24–26].

Multivariate analysis indicated that patients categorised as high-risk had a disproportionate risk of hospitalisation. However, high-risk individuals who had also been vaccinated had a lower risk of hospitalisation than individuals who were high-risk alone. Similarly, patients who were high-risk had a higher probability of >1 GP visit, whereas patients who were high-risk and vaccinated had a lower probability of >1 GP visit. These findings suggest that vaccination may help to reduce the risk of hospitalisation and additional GP visits in high-risk groups. This demonstrates the clear benefit of vaccination of high-risk individuals and the rationale for ensuring that coverage in this group of individuals be reinforced, as coverage in those younger than 65 years is below target. Low vaccination coverage places individuals at risk of serious complications and death because they are not being vaccinated against influenza. However, although our results show clearly that vaccinated high-risk patients in the database had lower rates of additional GP visits and hospitalisations than unvaccinated high-risk patients in the database, there may be potential for confounding by differences in health-seeking behaviour that could not be captured in the database or accounted for in the analysis. For example, surveys in adults with asthma [27] and parents of high-risk children [28] at single UK GP practices have identified lack of awareness, barriers to access and health beliefs as factors influencing influenza vaccine uptake. Such factors could have the potential to confound our results if they also affect patients’ readiness to seek treatment (for example, patients who wait until severe symptoms develop before presenting for medical attention may require higher levels of intervention than patients who present early). Our analysis would not be able to identify such potential confounding factors, and this is a limitation of the study. A recent case-control study using GPRD data in people at low risk for influenza in the UK found that influenza vaccination reduced the risk of influenza (OR 0.74, 95% CI 0.60, 0.91) when given within 4 months before seasonal influenza outbreaks [29]. However, despite the authors’ attempt to minimise confounding, they identified a residual confounding effect in that a group of people in the study with annual vaccinations in multiple years were also consistently more likely to develop influenza than those who received fewer annual vaccinations [29]. This may indicate that this group had predisposing medical conditions that made them simultaneously more likely to seek influenza vaccination and also at higher risk of influenza.

As per our assumption that the circulation data from the HPA was reflective of the strain circulation in the community, which is a limitation of our study, influenza B GP visits, hospitalisations and deaths showed peaks in influenza seasons with high influenza B activity and vaccine mismatch. The correlation coefficients show that for influenza B, deaths were more closely correlated with hospitalisations than hospitalisations with GP visits. For influenza A and other respiratory infections, hospitalisations were more closely correlated with GP visits than deaths with hospitalisation. The multivariate analysis also showed a statistically significant increase in the odds of hospitalisation in years with vaccine influenza B mismatch. In seasons with a mismatch between the vaccine lineage and the circulating influenza lineages, individuals may receive less clinical benefit from vaccination than anticipated, because the vaccine provides limited or no protection against the mismatched virus lineage. This suggests that improving vaccine protection against influenza B in mismatched years could be of value. Quadrivalent influenza vaccination provides protection against both influenza B lineages, which have started to co-circulate in recent years, and should therefore improve protection.

It should be noted that the mismatch analysis depends on an implicit assumption that the HPA surveillance data are representative of cases of respiratory infection seen in GP practices and hospitals nationally. However, the HPA data are derived from sentinel practices, which may differ from the more general pool of GP practices from which the GPRD data are derived. It is possible that using the HPA data to attribute cases between the various respiratory viruses could over- or under-estimate the proportion of infections due to each pathogen, and this is a limitation of the study.

A further limitation of the study is that complication status was assessed according to whether complications were recorded in the database. If any patients had complications that were not recorded, they could potentially have been mis-coded as having influenza without complications. To explore this possibility we investigated the full records of the small number of patients who were admitted to hospital despite having no complications of influenza recorded in the database. This showed that although the patients had no secondary diagnosis codes (and were therefore classed as having no complications), they in fact had complications such as pneumonia or other respiratory manifestations. Coding errors such as these form a natural limitation to any retrospective database study, as even a comprehensive database cannot capture all possible information.

Our data are likely to underestimate the true burden of ILI in the UK, because the years included in the study all happened to be years of low influenza activity, as presented in the primary publication (Watkins et al., in preparation). Our data would also not have identified hospital-acquired influenza infections. Furthermore, previous research has indicated that hospitalisation for influenza may be a trigger for long-term health decline in elderly patients [30]. Our study has not attempted to take such effects into account.

This study provides the most detailed quantitative data to date on the ILI burden in the UK. It should help to narrow the considerable uncertainties in modelling the potential impact of interventions [10].

## Conclusions

Our results indicate that although vaccination rates in target groups have increased over the study period, a substantial proportion of people are still not receiving influenza vaccination. Improving the uptake of influenza vaccination among elderly people and other high-risk groups has the potential to achieve substantial health benefits.

## Supporting Information

S1 File READ codes and ICD-10 codes. (DOCX)

S1 Table Absolute values for resource use and cost in low-risk and high-risk patients. (DOC)

S2 Table Resource use and cost in low-risk patients, inpatient admissions by route. (DOC)

S3 Table Resource use and cost in high-risk patients, inpatient admissions by route. (DOC)
