# Correction: Burden of Illness in UK Subjects with Reported Respiratory Infections Vaccinated or Unvaccinated against Influenza: A Retrospective Observational Study

**DOI:** 10.1371/journal.pone.0140719

**Published:** 2015-10-09

**Authors:** The PLOS ONE Staff

There is an error in the Funding section. The publisher apologizes for the error. The correct Funding information is as follows: This work was supported by GlaxoSmithKline Biologicals SA, which was involved in all stages of the study conduct and analysis and also funded preparation of the manuscript and page charges. All authors had full access to the data and agreed with the submission of the publication.

The Supporting Information files have been corrected to remove track changes markup. Please view the correct Supporting Information files here.

## Supporting Information

S1 File READ codes and ICD-10 codes. (DOCX)

S1 Table Absolute values for resource use and cost in low-risk and high-risk patients. (DOC)

S2 Table Resource use and cost in low-risk patients, inpatient admissions by route. (DOC)

S3 Table Resource use and cost in high-risk patients, inpatient admissions by route. (DOC)
