# Supplementary material for: Burden of Illness in UK Subjects with Reported Respiratory Infections Vaccinated or Unvaccinated against Influenza: A Retrospective Observational Study
Source: PLoS One. 2015 Aug 19;10(8):e0134928. doi: 10.1371/journal.pone.0134928 (PMC4546056; doi:10.1371/journal.pone.0134928)
Supplement: S1 Table — (DOC) [file pone.0134928.s002.doc]

**Burden of illness in UK subjects with reported respiratory infections vaccinated or unvaccinated against influenza: a retrospective observational study**

Rhys D. Pockett^1^, John Watkins^2^, Phil McEwan^1^, Genevieve Meier ^3^

^1^Swansea Center for Health Economics, Swansea University, SA2 8PP, Wales, United Kingdom

^2^College of Biomedical and Life Sciences, Cardiff University, Cardiff, CF14 4YS, Wales, United Kingdom

^3^Health Economics, GSK Vaccines, 1300, Wavre, Belgium

**S2 Table. Absolute values for resource use and cost** in low-risk and high-risk patients

|  | **Vaccinated** | | | **Non-Vaccinated** | | | | |
| --- | --- | --- | --- | --- | --- | --- | --- | --- |
|  | **Overall Influenza** | **Influenza with complications recorded** | **Influenza without complications recorded** | **Overall Influenza** | **Influenza with complications recorded** | | **Influenza without complications recorded** | |
|  | **N** | **N** | **N** | **N** | **N** | | **N** | |
| **Low-risk patients** |  |  |  |  | |  | |  |
| **Inpatient Admissions (All)** |  |  |  |  | |  | |  |
| Absolute number of admissions | 397 | 397 | - | 574 | | 565 | | 9 |
| Absolute number of admissions (UK) | 14,399 | 14,399 | - | 20,818 | | 20,492 | | 326 |
| Absolute length of stay | 5,138 | 5,138 | - | 4,398 | | 4,370 | | 28 |
| Absolute length of stay (UK) | 186,350 | 186,350 | - | 159,511 | | 158,496 | | 1,016 |
| Total absolute cost | £3,524,668 | £3,524,668 | - | £3,017,028 | | £2,997,820 | | £19,208 |
| Total absolute cost (UK) | £127,836,164 | £127,836,164 | - | £109,424,572 | | £108,727,917 | | £696,655 |
| **GP Surgery Visits** |  |  |  |  | |  | |  |
| Absolute No of Visits | 20,338 | 2,512 | 17,826 | 130,414 | | 11,422 | | 118,992 |
| Absolute No of Visits (UK) | 737,639 | 91,108 | 646,531 | 4,729,985 | | 414,264 | | 4,315,720 |
| Total Absolute cost | £732,168 | £90,432 | £641,736 | £4,694,904 | | £411,192 | | £4,283,712 |
| Total Absolute cost (UK) | £26,554,997 | £3,279,878 | £23,275,119 | £170,279,447 | | £14,913,520 | | £155,365,926 |
| **GP Prescriptions** |  |  |  |  | |  | |  |
| Absolute No of Prescriptions | 41,201 | 4,100 | 37,101 | 86,602 | | 9,381 | | 77,221 |
| Absolute No of Prescriptions (UK) | 1,494,319 | 148,703 | 1,345,616 | 3,140,967 | | 340,239 | | 2,800,728 |
| **Out Patient Clinic Care** |  |  |  |  | |  | |  |
| Absolute No of OP Visits | 95 | 33 | 62 | 324 | | 96 | | 228 |
| Absolute No of OP Visits (UK) | 3,446 | 1,197 | 2,249 | 11,751 | | 3,482 | | 8,269 |
| Total Absolute cost | £13,965 | £4,851 | £9,114 | £47,628 | | £14,112 | | £33,516 |
| Total Absolute cost (UK) | £506,497 | £175,941 | £330,556 | £1,727,420 | | £511,828 | | £1,215,592 |
| **High-risk patients** |  |  |  |  | |  | |  |
| **Inpatient Admissions (All)** |  |  |  |  | |  | |  |
| Absolute number of admissions | 628 | 628 | - | 257 | | 254 | | 3 |
| Absolute number of admissions (UK) | 22,777 | 22,777 | - | 9,321 | | 9,212 | | 109 |
| Absolute length of stay | 7,604 | 7,604 | - | 2,979 | | 2,967 | | 12 |
| Absolute length of stay (UK) | 275,789 | 275,789 | - | 108,045 | | 107,610 | | 435 |
| Total absolute cost | £5,216,344 | £5,216,344 | - | £2,043,594 | | £2,035,362 | | £8,232 |
| Total absolute cost (UK) | £189,191,551 | £189,191,551 | - | £74,119,099 | | £73,820,533 | | £298,566 |
| **GP surgery visits** |  |  |  |  | |  | |  |
| Absolute No of Visits | 16,116 | 3,391 | 12,725 | 11,436 | | 2,212 | | 9,224 |
| Absolute No of Visits (UK) | 584,511 | 122,988 | 461,523 | 414,772 | | 80,227 | | 334,545 |
| Total Absolute cost | £580,176 | £122,076 | £458,100 | £411,696 | | £79,632 | | £332,064 |
| Total Absolute cost (UK) | £21,042,400 | £4,427,574 | £16,614,826 | £14,931,800 | | £2,888,173 | | £12,043,627 |
| **GP Prescriptions** |  |  |  |  | |  | |  |
| Absolute No of Prescriptions | 58,167 | 8,800 | 49,367 | 23,190 | | 3,589 | | 19,601 |
| Absolute No of Prescriptions (UK) | 2,109,659 | 319,167 | 1,790,491 | 841,078 | | 130,169 | | 710,909 |
| **Out Patient Clinic Care** |  |  |  |  | |  | |  |
| Absolute No of OP Visits | 117 | 47 | 70 | 82 | | 29 | | 53 |
| Absolute No of OP Visits (UK) | 4,243 | 1,705 | 2,539 | 2,974 | | 1,052 | | 1,922 |
| Total Absolute cost | £17,199 | £6,909 | £10,290 | £12,054 | | £4,263 | | £7,791 |
| Total Absolute cost (UK) | £623,790 | £250,582 | £373,208 | £437,186 | | £154,615 | | £282,572 |

GP, general practitioner; OP, outpatient; SD, standard deviation; UK, extrapolated to UK population
