# Supplementary material for: Burden of Illness in UK Subjects with Reported Respiratory Infections Vaccinated or Unvaccinated against Influenza: A Retrospective Observational Study
Source: PLoS One. 2015 Aug 19;10(8):e0134928. doi: 10.1371/journal.pone.0134928 (PMC4546056; doi:10.1371/journal.pone.0134928)
Supplement: S2 Table — (DOC) [file pone.0134928.s003.doc]

**Burden of illness in UK subjects with reported respiratory infections vaccinated or unvaccinated against influenza: a retrospective observational study**

Rhys D. Pockett^1^, John Watkins^2^, Phil McEwan^1^, Genevieve Meier ^3^

^1^Swansea Center for Health Economics, Swansea University, SA2 8PP, Wales, United Kingdom

^2^College of Biomedical and Life Sciences, Cardiff University, Cardiff, CF14 4YS, Wales, United Kingdom

^3^Health Economics, GSK Vaccines, 1300, Wavre, Belgium

**S3 Table. Resource use and cost in low-risk patients, inpatient admissions by route**

|  | **Vaccinated** | | | | | | **Non-Vaccinated** | | | | | |
| --- | --- | --- | --- | --- | --- | --- | --- | --- | --- | --- | --- | --- |
|  | **Overall Influenza** | | **Influenza with complications recorded** | | **Influenza without complications recorded** | | **Overall Influenza** | | **Influenza with complications recorded** | | **Influenza without complications recorded** | |
|  | **N** | **%** | **N** | **%** | **N** | **%** | **N** | **%** | **N** | **%** | **N** | **%** |
| **Inpatient Admissions (via A&E)** |  |  |  |  |  |  |  |  |  |  |  |  |
| Had ≥ 1 hospital admission | 193 | 1.1% | 193 | 1.1% | 0 | 0.0% | 281 | 0.2% | 278 | 0.2% | 3 | 0.0% |
| Had ≥ 1 hospital admission (UK) | 7,000 |  | 7,000 |  | 0 |  | 10,192 |  | 10,083 |  | 109 |  |
| Absolute number of admissions | 210 |  | 210 |  | - |  | 298 |  | 295 |  | 3 |  |
| Absolute number of admissions (UK) | 7,616 |  | 7,616 |  | - |  | 10,808 |  | 10,699 |  | 109 |  |
| Mean number of unique admissions (SD) | 1.1 | (0.33) | 1.1 | (0.33) | - | - | 1.1 | (0.25) | 1.1 | (0.25) | 1 | (0.0) |
| Absolute length of stay | 3187 |  | 3187 |  | - |  | 2436 |  | 2421 |  | 15 |  |
| Absolute length of stay (UK) | 115,589 |  | 115,589 |  | - |  | 88,351 |  | 87,807 |  | 544 |  |
| Mean length of stay (SD) | 15.8 | (18.8) | 15.8 | (18.8) | - | - | 8.2 | (11.4) | 8.2 | (11.5) | 5 | (3.6) |
| Total absolute cost | £2,186,282 |  | £2,186,282 |  | - |  | £1,671,096 |  | £1,660,806 |  | £10,290 |  |
| Total absolute cost (UK) | £79,294,250 |  | £79,294,250 |  | - |  | £60,608,971 |  | £60,235,763 |  | ££373,208 |  |
| Mean total cost (SD) | £2,301,077 | £821,397 | £2,301,077 | £821,397 | - | - | £1,738,749 | £549,383 | £1,720,186 | £548,286 | £10,290 | £0 |
| Mean total cost (SD) (UK) | £83,457,749 | £29,791,243 | £83,457,749 | £29,791,243 | - | - | £63,062,678 | £19,925,569 | £62,389,416 | £19,885,782 | £373,208 | £0 |
| **Inpatient Admissions (via GP referral)** |  |  |  |  |  |  |  |  |  |  |  |  |
| Had ≥ 1 hospital admission | 113 | 0.6% | 113 | 0.6% | 0 | 0.0% | 201 | 0.2% | 196 | 0.2% | 5 | 0.0% |
| Had ≥ 1 hospital admission (UK) | 4,098 |  | 4,098 |  | 0 |  | 7,290 |  | 7,109 |  | 181 |  |
| Absolute number of admissions | 138 |  | 138 |  | - |  | 210 |  | 205 |  | 5 |  |
| Absolute number of admissions (UK) | 5,005 |  | 5,005 |  | - |  | 7,616 |  | 7,435 |  | 181 |  |
| Mean number of unique admissions (SD) | 1.2 | (0.64) | 1.2 | (0.64) | - | - | 1 | (0.21) | 1 | (0.21) | 1 | (0.0) |
| Absolute length of stay | 1477 |  | 1477 |  | - |  | 1534 |  | 1522 |  | 12 |  |
| Absolute length of stay (UK) | 53,569 |  | 53,569 |  | - |  | 55,637 |  | 55,201 |  | 435 |  |
| Mean length of stay (SD) | 11.4 | (13.6) | 11.4 | (13.6) | - | - | 7.1 | (10.1) | 7.2 | (10.1) | 2.4 | (3.1) |
| Total absolute cost | £1,013,222 |  | £1,013,222 |  | - |  | £1,052,324 |  | £1,044,092 |  | £8,232 |  |
| Total absolute cost (UK) | £36,748,543 |  | £36,748,543 |  | - |  | £38,166,733 |  | £37,868,167 |  | £298,566 |  |
| Mean total cost (SD) | £1,060,446 | £674,717 | £1,060,446 | £674,717 | - | - | £978,991 | £292,456 | £968,083 | £285,181 | £8,232 | £0 |
| Mean total cost (SD) (UK) | £38,461,310 | £24,471,307 | £38,461,310 | £24,471,307 | - | - | £35,507,019 | £10,607,085 | £35,111,397 | £10,343,228 | £298,566 | £0 |
| **Inpatient Admissions (via Other Route)** |  |  |  |  |  |  |  |  |  |  |  |  |
| Had ≥ 1 hospital admission | 36 | 0.2% | 36 | 0.2% | 0 | 0.0% | 52 | 0.0% | 51 | 0.0% | 1 | 0.0% |
| Had ≥ 1 hospital admission (UK) | 1,306 |  | 1,306 |  | 0 |  | 1,886 |  | 1,850 |  | 36 |  |
| Absolute number of admissions | 49 |  | 49 |  | - |  | 66 |  | 65 |  | 1 |  |
| Absolute number of admissions (UK) | 1,777 |  | 1,777 |  | - |  | 2,394 |  | 2,357 |  | 36 |  |
| Mean number of unique admissions (SD) | 1.4 | (0.64) | 1.4 | (0.64) | - | - | 1.3 | (0.49) | 1.3 | (0.49) | 1 | - |
| Absolute length of stay | 474 |  | 474 |  | - |  | 428 |  | 427 |  | 1 |  |
| Absolute length of stay (UK) | 17,192 |  | 17,192 |  | - |  | 15,523 |  | 15,487 |  | 36 |  |
| Mean length of stay (SD) | 11 | (11.2) | 11 | (11.2) | - | - | 7.2 | (7.5) | 7.3 | (7.5) | 1 | - |
| Total absolute cost | £325,164 |  | £325,164 |  | - |  | £293,608 |  | £292,922 |  | £686 |  |
| Total absolute cost (UK) | £11,793,371 |  | £11,793,371 |  | - |  | £10,648,867 |  | £10,623,986 |  | £24,881 |  |
| Mean total cost (SD) | £380,318 | £177,021 | £380,318 | £177,021 | - | - | £333,890 | £131,095 | £332,017 | £128,574 | £686 | - |
| Mean total cost (SD) (UK) | £13,793,751 | £6,420,374 | £13,793,751 | £6,420,374 | - | - | £12,109,855 | £4,754,684 | £12,041,923 | £4,663,250 | £24,881 | - |

A&E, accident and emergency; GP, general practitioner; SD, standard deviation; UK, extrapolated to UK population
