# Supplementary material for: Burden of Illness in UK Subjects with Reported Respiratory Infections Vaccinated or Unvaccinated against Influenza: A Retrospective Observational Study
Source: PLoS One. 2015 Aug 19;10(8):e0134928. doi: 10.1371/journal.pone.0134928 (PMC4546056; doi:10.1371/journal.pone.0134928)
Supplement: S3 Table — (DOC) [file pone.0134928.s004.doc]

**Burden of illness in UK subjects with reported respiratory infections vaccinated or unvaccinated against influenza: a retrospective observational study**

Rhys D. Pockett^1^, John Watkins^2^, Phil McEwan^1^, Genevieve Meier ^3^

^1^Swansea Center for Health Economics, Swansea University, SA2 8PP, Wales, United Kingdom

^2^College of Biomedical and Life Sciences, Cardiff University, Cardiff, CF14 4YS, Wales, United Kingdom

^3^Health Economics, GSK Vaccines, 1300, Wavre, Belgium

**S4 Table. Resource use and cost in high-risk patients, inpatient admissions by route**

|  | **Vaccinated** | | | | | | **Non-Vaccinated** | | | | | |
| --- | --- | --- | --- | --- | --- | --- | --- | --- | --- | --- | --- | --- |
|  | **Overall Influenza** | | **Influenza with complications recorded** | | **Influenza without complications recorded** | | **Overall Influenza** | | **Influenza with complications recorded** | | **Influenza without complications recorded** | |
|  | **N** | **%** | **N** | **%** | **N** | **%** | **N** | **%** | **N** | **%** | **N** | **%** |
| **Inpatient Admissions (via A&E)** |  |  |  |  |  |  |  |  |  |  |  |  |
| Had ≥ 1 hospital admission | 300 | 2.4% | 300 | 2.4% | 0 | 0.0% | 134 | 1.5% | 133 | 1.5% | 1 | 0.0% |
| Had ≥ 1 hospital admission (UK) | 10,881 |  | 10,881 |  | 0 |  | 4,860 |  | 4,824 |  | 36 |  |
| Absolute number of admissions | 353 |  | 353 |  | - |  | 151 |  | 150 |  | 1 |  |
| Absolute number of admissions (UK) | 12,803 |  | 12,803 |  | - |  | 5,477 |  | 5,440 |  | 36 |  |
| Mean number of unique admissions (SD) | 1.2 | (0.45) | 1.2 | (0.45) | - | - | 1.1 | (0.48) | 1.1 | (0.48) | 1 | - |
| Absolute length of stay | 4,696 |  | 4,696 |  | - |  | 1,854 |  | 1,851 |  | 3 |  |
| Absolute length of stay (UK) | 170,319 |  | 170,319 |  | - |  | 67,243 |  | 67,134 |  | 109 |  |
| Mean length of stay (SD) | 14.1 | (17.8) | 14.1 | (17.8) | - | - | 13.2 | (22.3) | 13.3 | (22.3) | 3 | - |
| Total absolute cost | £3,221,456 |  | £3,221,456 |  | - |  | £1,271,844 |  | £1,269,786 |  | £2,058 |  |
| Total absolute cost (UK) | £116,838,970 |  | £116,838,970 |  | - |  | £46,128,503 |  | £46,053,861 |  | £74,642 |  |
| Mean total cost (SD) | £3,482,136 | £1,648,458 | £3,482,136 | £1,648,458 | - | - | £1,334,736 | £983,954 | £1,334,812 | £976,612 | £2,058 | - |
| Mean total cost (SD) (UK) | £126,293,571 | £59,787,914 | £126,293,571 | £59,787,914 | - | - | £48,409,532 | £35,687,022 | £48,412,289 | £35,420,735 | £74,642 |  |
| **Inpatient Admissions (via GP referral)** |  |  |  |  |  |  |  |  |  |  |  |  |
| Had ≥ 1 hospital admission | 153 | 1.2% | 153 | 1.2% | 0 | 0.0% | 65 | 0.7% | 63 | 0.7% | 2 | 0.0% |
| Had ≥ 1 hospital admission (UK) | 5,549 |  | 5,549 |  | 0 |  | 2,357 |  | 2,285 |  | 73 |  |
| Absolute number of admissions | 191 |  | 191 |  | - |  | 80 |  | 78 |  | 2 |  |
| Absolute number of admissions (UK) | 6,927 |  | 6,927 |  | - |  | 2,902 |  | 2,829 |  | 73 |  |
| Mean number of unique admissions (SD) | 1.2 | (0.53) | 1.2 | (0.53) | - | - | 1.2 | (0.52) | 1.2 | (0.53) | 1 | (4.5) |
| Absolute length of stay | 2,195 |  | 2,195 |  | - |  | 846 |  | 837 |  | 9 |  |
| Absolute length of stay (UK) | 79,610 |  | 79,610 |  | - |  | 30,684 |  | 30,357 |  | 326 |  |
| Mean length of stay (SD) | 11.9 | (13.9) | 11.9 | (13.9) | - | - | 11.4 | (16.0) | 11.6 | (16.2) | 4.5 | (4.9) |
| Total absolute cost | £1,505,770 |  | £1,505,770 |  | - |  | £580,356 |  | £574,182 |  | £6,174 |  |
| Total absolute cost (UK) | £54,612,764 |  | £54,612,764 |  | - |  | £21,048,928 |  | £20,825,004 |  | £223,925 |  |
| Mean total cost (SD) | £1,498,800 | £773,226 | £1,498,800 | £773,226 | - | - | £609,991 | £370,989 | £601,595 | £371,070 | £6,174 | £30,253 |
| Mean total cost (SD) (UK) | £54,359,969 | £28,044,129 | £54,359,969 | £28,044,129 | - | - | £22,123,760 | £13,455,398 | £21,819,246 | £13,458,336 | £223,925 | £1,097,246 |
| **Inpatient Admissions (via Other Route)** |  |  |  |  |  |  |  |  |  |  |  |  |
| Had ≥ 1 hospital admission | 50 | 0.4% | 50 | 0.4% | 0 | 0.0% | 23 | 0.3% | 23 | 0.3% | 0 | 0.0% |
| Had ≥ 1 hospital admission (UK) | 1,813 |  | 1,813 |  | 0 |  | 834 |  | 834 |  | 0 |  |
| Absolute number of admissions | 84 |  | 84 |  | - |  | 26 |  | 26 |  | - |  |
| Absolute number of admissions (UK) | 3,047 |  | 3,047 |  | - |  | 943 |  | 943 |  | - |  |
| Mean number of unique admissions (SD) | 1.7 | (0.74) | 1.7 | (0.74) | - | - | 1.1 | (0.63) | 1.1 | (0.63) | - | - |
| Absolute length of stay | 713 |  | 713 |  | - |  | 279 |  | 279 |  | - |  |
| Absolute length of stay (UK) | 25,860 |  | 25,860 |  | - |  | 10,119 |  | 10,119 |  | - |  |
| Mean length of stay (SD) | 8.7 | (6.2) | 8.7 | (6.2) | - | - | 10.7 | (7.8) | 10.7 | (7.8) | - | - |
| Total absolute cost | £489,118 |  | £489,118 |  | - |  | £191,394 |  | £191,394 |  | - |  |
| Total absolute cost (UK) | £17,739,818 |  | £17,739,818 |  | - |  | £6,941,668 |  | £6,941,668 |  |  |  |
| Mean total cost (SD) | £507,297 | £157,368 | £507,297 | £157,368 | - | - | £185,707 | £77,533 | £185,707 | £77,533 | - | - |
| Mean total cost (SD) (UK) | £18,399,152 | £5,707,579 | £18,399,152 | £5,707,579 | - | - | £6,735,406 | £2,812,044 | £6,735,406 | £2,812,044 | - | - |

A&E, accident and emergency; GP, general practitioner; SD, standard deviation; UK, extrapolated to UK population
